# Expression of inflammatory interleukins and selected miRNAs in non-small cell lung cancer

**DOI:** 10.1038/s41598-021-84408-1

**Published:** 2021-03-03

**Authors:** Agata Dutkowska, Bartosz Szmyd, Marcin Kaszkowiak, Daria Domańska-Senderowska, Dorota Pastuszak-Lewandoska, Ewa Brzeziańska-Lasota, Jacek Kordiak, Adam Antczak

**Affiliations:** 1 Department of General and Oncological Pulmonology, Medical University of Lodz, Lodz, Poland; 2 Department of Biomedicine and Genetics, Medical University of Lodz, Lodz, Poland; 3 Department of Microbiology and Laboratory Medical Immunology, Lodz, Poland; 4 Department of Chest Surgery, General and Oncological Surgery, University Teaching Hospital No. 2, Medical University of Lodz, Lodz, Poland

**Keywords:** Cancer, Genetics, Immunology

## Abstract

Tumours are characterised by an ability to avoid immune destruction and the presence of cancer-associated inflammation. Better understanding of the link between lung cancer and such inflammation is vital for early detection and personalized treatment. Thus, we examined the mRNA expression of interleukins *IL-1β, IL-6, IL-17* and *miR-9, miR-122* as potential useful biomarkers of NSCLC. Tumour tissues, non-cancerous tissue and blood samples were collected from 39 patients with primary NSCLC undergoing surgical treatment. The selected RNA was isolated from tissue samples and selected miRNAs from peripheral blood exosomes. This RNA was transcribed to cDNA and quantified using RT-qPCR. Significantly higher expression of the selected interleukins was observed in non-cancerous than tumour tissue, and *IL-6* was significantly higher in the tumour tissue of patients with a history of ≤ 40 pack-years (PYs) (2.197, IQR: 0.821–4.415) than in those with > 40 PYs (0.461, IQR: 0.372–0.741; p = 0.037). It is clear that inflammatory processes play a role in NSCLC, as indicated by the upregulation of *IL-1β* and *IL-6* in tumour and adjacent tissue, and that smoking has a strong influence on inflammation in tumourigenesis, demonstrated by the upregulation of *IL-6* in tumour samples among patients with ≤ 40 PYs compared to > 40 PYs.

## Introduction

Cancer is one of the greatest challenges faced by modern medicine. In 2018 about 1 700 000 new cancer cases and 610 000 cancer-related deaths were recorded in United States alone^1^. Lung cancer (LC) was responsible for, respectively, 13.5% and 25.3% of those values, making it the second-most prevalent carcinoma and that with the greatest morbidity. The most popular LC subtype is that of non-small cell lung cancer (NSCLC) (85% of cases)^2^. This can be further divided to adenocarcinoma (AC), squamous-cell carcinoma (SCC), and large cell carcinoma. The mean 5-year LC survival rate is estimated at around 20%^3^, which is far from satisfying. This low survival rate is primarily due to relatively late diagnosis caused by lack of screening and specific symptoms.

There is clearly an urgent need for new diagnostic and treatment strategies. However, these require a deeper understanding of the underlying mechanisms and interactions associated with cancer. Inflammation is a crucial process associated with lung cancer. It is related to two out of ten hallmarks of cancer in general: avoiding immune destruction and tumour-promoting inflammation^4^. Cancer-related inflammation is an especially interesting area of study, as recently-developed immunotherapy strategies, intended to use the immune system to fight cancer, require an understanding of the mechanisms of immune response.

The need to identify non-invasive diagnostic markers based on molecular targets has fostered the growth of various studies examining the potential of miRNA. These small strands of ribonucleic acid, up to 30 bases in length, are responsible for regulating the expression of many genes, including key cancer immune-modulators. Their proper functioning is crucial for maintaining correct cell activity^5^, and any disruption in their regulatory activity may result in the development of a number of serious diseases, including cancer^6,7^. The present study analyses the expression of selected genes and miRNAs associated with inflammatory processes to better understand their contribution to cancer-related inflammation:

- IL-1β—secreted by macrophages. An important mediator of inflammatory response but also responsible for cell proliferation, differentiation and apoptosis^8,9^.
- IL-6—a multifunctional cytokine which takes part in B-cells maturation, among other things. It acts as an endogenous pyrogen secreted at inflammatory sites^10^.
- IL-17—a pro-inflammatory cytokine secreted by activated T lymphocytes. It is associated with chronic inflammation^11^.

Their expression may be regulated by miRNAs *miR-9* and *miR-122,* which are considered to have inflammation-related properties. For example, *miR-9* is reported to inhibit inflammatory processes by targeting *IL-6* mRNA^12^. Many studies suggest that *miR-9* plays an important role in maintaining the balance between inflammation and lymphangiogenesis^13^, inhibition of erythropoiesis^14^ and in suppressing the proliferation of breast cancer cells^15^. It is also involved in lung cancer cell invasion and adhesion^16^. Hence, *miR-9* expression has a significant influence on the course of LC in terms of inflammatory response. In addition, *miR-122* affects expression level of both *IL-1β*^17^ and *IL-6*^18^ as well as is believed to impact a range of cancer-related processes, such as inhibiting metastatic processes in lung cancer^19^ and promoting cell proliferation and invasion in renal cell carcinoma^20^. In addition to its influence on the cell cycle, it also affects glucose metabolism in breast cancer^21^ and inflammation in the liver^22^. Very few studies have examined the influence of *miR-122* on inflammatory processes in lung cancer.

## Results

### Selected genes

**Figure 1:**
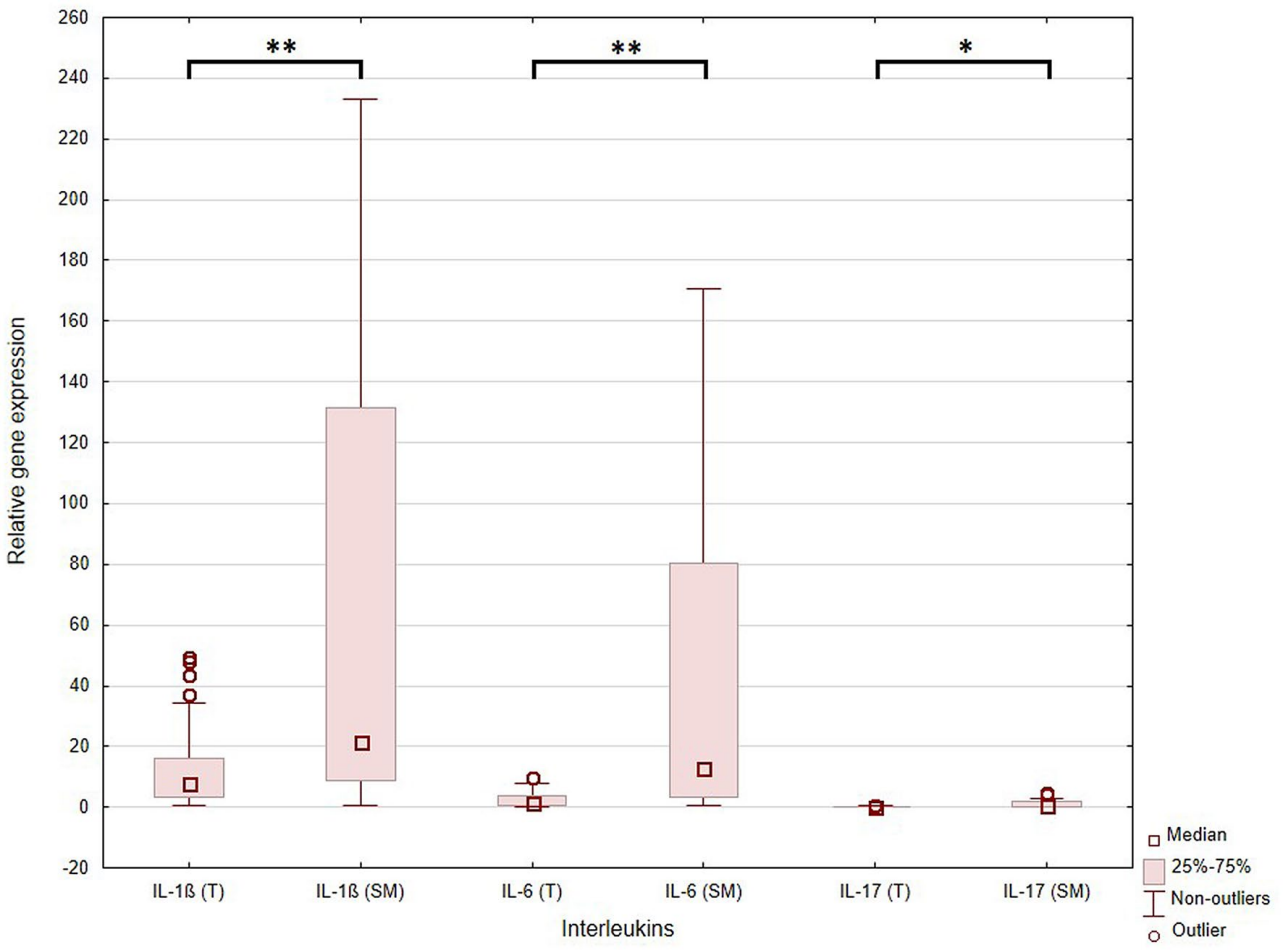
The relative expression of the examined interleukins*: IL-1β*, *IL-6*, and *IL-17* assessed in the tumour tissue sample (T) and in the surgical margin (SM). The dependences between each interleukin in both samples were statistically significant: *p = 0.008, **p < 0.001 (Wilcoxon test). Relative expression (RQ) value, adjusted to *ACTB* gene (endogenous control) expression level.

The relative expression of a number of selected genes (RQ) was evaluated for a group of 39 patients. As the obtained RQ values were not normally distributed (Shapiro–Wilk test, p < 0.05), non-parametric tests were used for statistical analyses. The RQ values of studied interleukins were significantly higher in the surgical margin than in tumour : *IL-1β* was 21.591 (IQR: 8.966–119.837) vs. 7.794 (IQR: 3.46–15.917), *IL-6* was 13.03 (IQR: 3.544–77.858) vs 1.273 (IQR: 0.437–3.488), and *IL-17* was 0.417 (IQR: 0.112–1.524) vs. 0.097 (IQR: 0.02–0.179). The p-values of Wilcoxon tests were < 0.001, < 0.001, and 0.008, respectively (see Fig. 1). Moreover, both *IL-1β* and *IL-6* were upregulated in the tumour sample and surgical margin in comparison to the calibrator (RQ > 1). *IL-17* was downregulated in comparison to the calibrator (RQ < 1).

The RQ value of the tested interleukins was found to be insignificantly higher RQ value among females than male patients. Only *IL-1β*, measured in tumour tissue, demonstrated insignificantly higher expression among male patients: 10.662 (IQR: 4.058–18.640) vs. 7.649 (IQR: 2.031–14.136). The highest RQ values, in both the tumour lesion and surgical margin, were observed among patients younger than 60 years old. However, these findings, as well as the correlations between age and the tested interleukins, were not statistically significant.

Interestingly, all analysed interleukins taken from tumour samples demonstrated upregulated expression in the squamous cell subtype compared to adenocarcinoma: ***IL-17:*** 0.135 (IQR: 0.043–0.212) vs. 0.018 (IQR: 0.012–0.143), (UMW test, p = 0.032); ***IL-1β:*** 9.302 (IQR: 3.839–16.335) vs. 3.831 (IQR: 1.095–12.565); ***IL-6:*** 1.608 (IQR: 0.455–4.074) vs 0.604 (IQR: 0.360–1.510). The same dependencies were observed in surgical margin samples: ***IL-1β:*** 21.617 (IQR:10.060–84.920) vs. 17.973 (IQR: 7.039–138.839); ***IL-6:*** 23.171 (5.000–80.318) vs. 2.580 (IQR: 1.615–12.430). These observations were not statistically significant.

In samples taken from the tumour tissue, the RQ values of *IL-1β* and *IL-6* were higher in patients with pT1 stage vs. pT3 stage, while those of *IL-17* were higher in pT3 vs. pT2 patients (see Table 1).

**Table 1:** Clinical and pathological features: median of expression level (RQ value) of evaluated genes.

Clinical and pathological features	N	*IL-1β* (tumour tissue)	*IL-1β* (surgical margin)	*p* value	*IL-6* (tumour tissue)	*IL-6* (surgical margin)	*p* value	*IL-17* (tumour tissue)	*IL-17* (surgical margin)	*p* value
Entire group	39	7.794	21.591	< 0.001	1.273	13.030	< 0.001	0.097	0.417	0.008
**Gender**										
Women	19	7.649	53.541	^1^0.271 ^2^0.240	1.273	31.343	^1^0.956 ^2^0.137	0.129	0.756	^1^0.631 ^2^0.137
Men	20	10.662	15.443		1.194	12.430		0.053	0.290	
**Age group**										
≤ 60	6	14.314	52.244	^1^0.757 ^2^0.310	1.702	46.374	^1^0.987 ^2^0.516	0.142	0.642	^1^0.673 ^2^0.107
60–70	20	5.307	21.592		0.821	21.361		0.066	0.428	
> 70	13	4.476	14.016		1.422	8.092		0.045	0.081	
**Histopathological type**										
AC	14	3.831	17.973	^1^0.183 ^2^0.859	0.604	2.580	^1^0.1204 ^2^0.0904	0.018	0.363	^1^0.461 ^2^0.032
SCC	25	9.302	21.617		1.608	23.171		0.135	0.681	
***pTNM***										
**Tumour size**										
pT1	5	10.169	12.912	^1^0.190 ^2^0.268	2.001	7.052	^1^0.333 ^2^0.143	0.147	0.350	^1^0.072 ^2^0.731
pT2	31	8.924	26.695		1.269	12.591		0.049	0.407	
pT3	3	2.122	68.718		0.438	145.324		0.249	0.843	
**Lymph nodes invasion**										
N0	33	6.063	21.617	^1^0.522 ^2^0.866	0.756	12.869	^1^0.258 ^2^0.998	0.060	0.517	^1^0.362 ^2^0.690
N1	3	5.307	12.913		2.197	23.171		0.164	0.244	
N2	3	12.376	74.450		3.910	12.752		0.185	1.212	
**AJCC**										
AJCC I	19	3.836	10.031	^1^0.302 ^2^0.080	0.604	4.098	^1^0.212 ^2^0.028	0.038	0.322	^1^0.111 ^2^0.362
AJCC II	17	6.624	68.718		1.635	65.388		0.137	0.799	
AJCC III	3	12.376	74.450		3.910	12.752		0.185	1.212	
**Pack years**										
≤ 40 PYs	20	12.376	68.7184	^1^0.281 ^2^0.200	2.197	32.581	^1^0.007 ^2^0.064	0.138	0.582	^1^0.525 ^2^0.422
> 40 PYs	14	4.476	12.966		0.461	5.001		0.033	0.353	
No data	5	–	–		–	–		–	–	

^1^Pertain to expression in tumour tissue.

^2^Pertain to expression in surgical margin.

With regard to cigarette smoking, significantly higher expression of *IL-6* was observed in the tumour tissue of patients with a history of ≤ 40 PYs, i.e. 2.197 (IQR: 0.821–4.415) than in those with > 40 PYs, i.e. 0.461 (IQR: 0.372–0.741), (UMW test, p = 0.037). *IL-1β* and *IL-17* expression was also higher in the tumour samples of taken from patients with a shorter smoking history, but only insignificantly. No statistically significant dependences were observed for familial risk of cancer.

### MicroRNA

MiRNA was assessed in pre- and postoperative blood samples (n_pre_ = 30 and n_post_ = 23, respectively). *MiR-9* was down-regulated in the postoperative samples regarding the calibrator while *miR-22* was up-regulated. The analysed miRNAs demonstrated similar levels of expression at both time points: i.e. before and after surgical treatment. Median *miR-9* expression was 0.726 (IQR: 0.479–1.002) before and 0.754 (IQR: 0.574–1.318) after surgery*;* median *miR-122* expression was 1.378 (IQR: 0.999–2.096) before and 1.327 (IQR: 0.764–1.747) after.

The RQ values of *miR-9* were insignificantly higher among male patients than female patients at both time-points: *miR-9* before operation was 0.866 (IQR: 0.626–1.104) for men vs. 0.603 (IQR: 0.385–0.819) for women (p = 0.089, UMW), with these values being 0.917 (IQR: 0.602–1.277) vs. 0.694 (IQR: 0.508–1.310) after the operation (p = 0.786, UMW). The opposite trend was noted for *miR-122.* No statistically significant relationships were observed between age and the expression of the selected miRNAs.

*MiR-9* was upregulated among patients with adenocarcinoma, and *miR-122* for those with squamous-cell carcinoma (see Table 2). These observations were not statistically significant. Expression analysis in relation to tumour size (T feature, according TNM staging) found *miR-9* expression to be greater in pT2 than pT3, both before and after surgery (RQ: 0.796 and RQ:0.917 respectively). Similarly, higher *MiR-122* levels were noted in pT3 than pT2, both before and after surgery (RQ: 1.773 and RQ: 2.398 respectively). No statistically significant relationship was found between lymph node invasion value /AJCC scale and miRNA expression. The clinical and pathological features of the tested miRNAs, with median expression (RQ value), are collected in Table 2. Cross results.

**Table 2:** Clinical and pathological features: median expression level (RQ value) of evaluated microRNA. ^1^pertain to expression before surgical treatment, ^2^pertain to expression after surgery.

Clinical and pathological features	N	*miR-9* (before surgery)	*miR-9* (after surgery)	*p* value	*miR-122* (before surgery)	*miR-122* (after surgery)	*p* value
Entire group	39	0.726	0.754	0.758	1.378	1.327	0.958
**Gender**							
Woman	19	0.602	0.694	^1^0.089 ^2^0.786	1.662	1.442	^1^0.089 ^2^0.786
Men	20	0.866	0.917		1.155	0.407	
**Age group**							
≤ 60	6	0.856	1.333	^1^0.772 ^2^0.190	1.170	0.758	^1^0.772 ^2^0.190
60–70	20	0.600	0.744		1.667	1.345	
> 70	13	0.904	0.601		1.109	1.664	
**Histopathological type**							
AC	14	0.796	0.973	^1^0.422 ^2^0.428	1.256	1.094	^1^0.422 ^2^0.428
SCC	25	0.710	0.754		1.409	1.327	
***pTNM***							
**Tumour size**							
pT1	5	0.671	0.831	^1^0.617 ^2^0.421	1.489	1.362	^1^0.617 ^2^0.421
pT2	31	0.796	0.917		1.256	1.127	
pT3	3	0.599	0.417		1.773	2.398	
**Lymph nodes invasion**							
N0	33	0.710	0.917	^1^0.162 ^2^1.000	1.409	1.127	^1^0.162 ^2^1.000
N1	3	1.314	0.547		0.779	1.828	
N2	3	0.581	–		1.722	–	
**AJCC**							
AJCC I	19	0.679	0.744	^1^0.413 ^2^1.000	1.473	1.345	^1^0.413 ^2^1.000
AJCC II	17	0.817	1.079		1.224	0.927	
AJCC III	3	0.581	–		1.722	–	
**Pack years**							
≤ 40 PYs	20	0.698	0.904	^1^0.982 ^2^0.705	1.433	1.169	^1^0.982 ^2^0.705
> 40 PYs	14	0.807	0.753		1.240	1.327	
No data	5	–	–		–	–	

**Figure 2:**
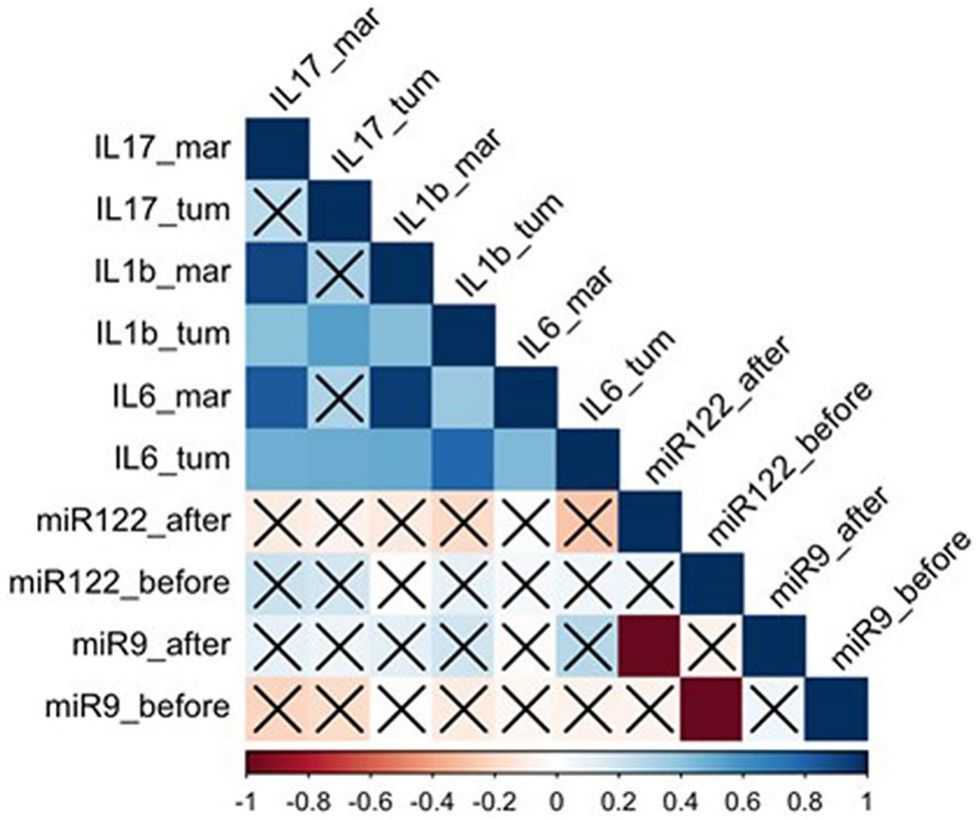
A Spearman’s rank correlogram. Correlations marked with X are not statistically significant (p > 0.05). The colour scale represents Spearman’s correlation coefficient (r). Legend: *tum* tumour sample; *mar* surgical margin.

No statistically significant correlations were observed between the expressions of the examined miRNAs (*miR-9, miR-122*) and interleukins (*IL-1β, IL-6,* and *IL-17*). More detailed results (R and p-values) are collected in Fig. 2.

## Discussion

One of the underlying causes of all forms of cancer, including lung cancer, is inflammation. As such, a greater understanding of inflammatory processes and their biochemical bases is crucial for the design of new diagnostic techniques and therapeutic strategies. One such approach is immunotherapy, a treatment strategy which has emerged from profound studies of the inflammatory process. The present study examines the relationship between inflammation markers and the miRNAs regulating them with the aim of identifying non-invasive markers of cancer disease progression.

The study focussed on the expression of pro-inflammatory interleukins in lung cancer tissue and in macroscopically-unchanged tissue taken from the surgical margin.

Our findings indicate significant upregulation of the examined interleukins (*IL-1β, IL-6* and *IL-17*) in the surgical margin in comparison to the tumour lesion. This observation may be related to epigenetic changes such as DNA methylation and histone modification occurring in the genes encoding the cytokines^23,24^. Observed reduction of *IL-1β* and *IL-6* expression at the genetic level in tumour lesion are consistent with those of previous studies. Tekpli et al. report lower expression of *IL-1β* and *IL-6* in both NSCLC tissue and in NSCLC cell lines, and attribute it to promoter methylation of these genes^25^.

### Link between *IL-1β* and LC

Our findings also indicate that of the studied cytokines, pro-inflammatory *IL-1β* demonstrated the highest expression in non-cancerous tissue*.* Interestingly, IL-1β is recognized as one of the most crucial cytokines in immunological regulation during tumour formation. Our observations confirm the results of other authors. Vikhreva et al. also observed *IL-1β* upregulation, however not in cancer tissue but in human NSCLC cell lines^26^. Additionally, many other studies focus on role of innate immune system in carcinogenesis, confirmed importance of *IL-1β* in lung cancer development^23,25–28^.

It was documented that chronic inflammatory conditions increase the risk of developing cancer^29^**.** We demonstrated that the *IL-1β* upregulation was more frequent in less advanced stages of lung cancer pT1 when compared with pT2 and pT3 + pT4. Cancer progression is associated with inflammatory and immunosuppressive processes. Myeloid-derived suppressor cells (MDSC) are produced in bone marrow under the influence of tumor cell derived substances. Macrophages present in the tumour microenvironment (tumour-associated macrophages, TAM), which produce IL-1β. It has been observed that implanted breast cancer tumours regress in mice deficient in IL-1β or in wild-type mice treated with anti–IL-1β antibodies. Such regression is caused by decreased tumour-related immunosuppression, meditated mainly by TAM, and increased anti-tumour immunity, mediated by elevated dendritic cell function and activated cytotoxic CD8 lymphocytes^30^. In our study, we confirmed the highest expression level of *IL-1B* in T1 compared to T3, this is possible due to the initial stage of carcinogenesis in response to chemotherapy. It should be pointed that regulation of *IL-1β* expression is depending on complex interactions between tumor cells and infiltrating host inflammatory cells like lymphocytes and macrophages. Anti-tumor defense mechanism, with the contribution of macrophages and lymphocytes in early stage of carcinogenesis, in the next phase leads to tumor progression or regression. Furthermore, *IL-1β* may be involved in the initial stages of carcinogenesis by activating infiltrating phagocytes or the target cells for transformation to produce mutagenic ROI or NOI. In effect, extensive proliferation leads to the accumulation of mutations in the pre-malignant cell^31^.

Lung AC and SCC demonstrate significant differences in key genomic, genetic, and clinical characteristics, including tumor differentiation, stage-specific survival, underlying drivers, and likely response to various therapies. Many authors suggested significant differences in ”immunologic signature” of each tumor subtype which has serious impact on treatment response.

In our study, the highest *IL-1β* expression was observed in SCC samples among NSCLC histotypes. However, in Kong et al*.* study, in the NSCLC, mRNA levels of *IL-1β*, were significantly higher in the AC than in the SCC histotypes^32^.

### Link between *IL-6* and LC

Another interleukin commonly linked with a cancerous inflammatory response is IL-6. Il-6 is a multidirectional interleukin secreted mostly by monocytes and macrophages. In our study, we showed higher expression of *IL-6* in surgical tissue margin compared to cancer tissue. Unfortunately, we can not refer our data to the results of others. According to our knowledge, there are no published data related to this kind of study It was documented that, IL-1 and IL-6 are involving in pro-inflammatory process, as complex interaction between tumour cells and host inflammatory system, leading to either to progression or regression of the tumour. Complex interaction between these cytokines in lung cancer is related to autocrine and/or paracrine mechanism. Therefore, the level of this cytokines in the blood in NSCLC patient is not always similar to the level of cytokine at the protein/mRNA level in the tissue. Moreover IL-1β also induced expression of IL-6 and other cytokines (e.g. GM-CSF, MCAF, TNF). Especially IL-6 is responsible for negative feedback of TNF secretion^33^. IL-6 plays a direct role in the prevention of apoptosis (deleting the genes involved in the cell cycle) and acts as an autocrine growth factor for tumours^34^. Studies have shown that IL-6 is associated with tumour progression in NSCLC^35^.

Yuquan et al. report high *IL-6* expression in NSCLC tissue in comparison to benign pulmonary lesions (BPL) and note interesting correlations between *EGFR* and IL‐6R signalling pathways^36^. High *IL-6* expression has also been reported in the blood of patients with NSCLC compared to healthy controls and was also found to be a marker of poor prognosis^37^. However, the level of such molecules in the blood of a cancer patient is not always identical to that in tissue, because their peripheral expression may be caused by other factors.

In the early stage of carcinogenesis, the immune system reacts vigorously to proliferating neoplasm cells. Later, as the disease progresses, tumor cells escape from immune surveillance and in effect a state of tumor-specific tolerance develops, hence the inflammatory cascade may be reduced in more advanced forms of the disease and at the epicenter of the tumor mass.

Our results demonstrate that *IL-6* upregulation was more frequent in less advanced stages of lung cancer (pT1) than in more advanced pT2 and pT3 + 4. Similar results were observed in adenocarcinoma of the lung, but these results were not statistically significant^38^.

In our study, *IL-6* upregulation was observed in SCC samples, with the highest frequency of increased gene expression observed among NSCLC histotypes. This is in line with Shintani et al. (2008), who report higher expression of IL-6 in SCC in cell lines, but the results were not statistically significant^39^. In contrast to the results of other investigators, Balabko et al. note the presence of complicated interactions between Th17 lymphocytes, IL-6R and the pSTAT3/BATF/RorγT-axis in adenocarcinoma as compared to SCC of the lung, and higher expression of IL-6R in AC than SCC^40^. Silva proposes that differences in immunological signalling exist between particular types of NSCLC and therefore in the expression of involved interleukins^37^.

The observed differences in cytokine expression in different NSCLC subtypes may be due to different distribution of individual immune cells in AC and SCC. Our observations may in future contribute to better understanding subtype-specific immune characteristics.

### Link between *IL-17* and LC

IL-17 is another important pro-inflammatory molecule, and one that plays a role in chronic inflammatory, autoimmune diseases and inflammation-associated tumours^41^. Many studies have shown that IL-17 is involved in the metastasis and progression of lung cancer by directly, or indirectly, promoting tumour angiogenesis and cell proliferation, and by inhibiting apoptosis.

In our study, *IL-17* also revealed higher expression in the surgical margin than the tumour sample. Unfortunately, again, no comparable studies exist in the current literature regarding the expression of inflammatory interleukins in the microenvironment and in NSCLC. As so far, the study focuses on IL-17 and its contribution in lung cancer development documented important role of IL-17 in metastasis and progression. Moreover, the higher expression of IL-17 in more advanced stages of NSCLC was documented^41^.

Similarly, Chen et al. note significantly increased expression of *IL-17* in NSCLC in patients with higher lymphatic vessel density^42^. Although our study group consisted of patients in operable TNM status, and so without distant metastases, patients with pT3 nevertheless presented higher expression of *IL-17* than those with pT1 stage. This data, to some extent, suggests that its elevated expression is related to higher lung cancer stage.

Our findings indicate significant upregulation of *IL-17* in SCC. Although no studies have analysed *IL-17* expression in NSCLC tissue study using similar methodology, some examine IL-17 protein level in the blood of patients with different histopathologic NSCLC subtypes^43^ and in NSCLC tissue^44^; they indicate no significant differences in expression between AC and SCC.

### MicroRNA as cancer biomarkers

Recently it has been confirmed that increased level of many classes of miRNA in plasma may act as an independent marker of diagnosis and survival in patients with lung cancer. MiRNAs are potentially easy to measure and obtain^45^. In our present study, we compared miRNA expression level in the blood of NSCLC patients at two time points: before and after NSCLC resection. The present study examines the levels of two miRNAs that are recognized as cytokine regulators: *miR-9* and *miR-122*.

### Role of miR-9 in NSCLC

The function of *miR-9* in lung cancer is unclear. However, it has been proposed that *miR-9* may regulate NSCLC cell invasion and migration via epithelial-mesenchymal transition (EMT) process in NSCLC cell lines^46^. It is proposed that *miR*-9 promotes a metastatic phenotype in lung cancer, due to the role of *miR-9* as the important EMT regulator. This function of *miR-9* has also been confirmed in other tumours, like breast cancer^15^. These findings imply that *miR-9* expression may be altered in advanced stages of lung cancer. Our findings confirmed a reduction in *miR-9* expression level in more advanced lung tumours. In contrast, Yang et al. found *miR-9* to be upregulated in NSCLC patients from a Chinese population compared to controls. At his study, more than half of the cohort presented with stage III or IV cancer according to AJCC, so with local or distant metastases^47^.

The analysis of use of *miR-9* expression level depending on the histopathological differentiation is poorly described in the medical literature. However, it is interesting to note that significantly higher expression of *miR-9* was observed in AC compared to SCC. The same observation was made by Yang et al.^47^ in the blood of NSCLC patients. In addition, significantly higher expression of *miR-9* has been noted in NSCLC tumour tissue compared to adjacent normal lung tissues^48^.

Our results documented an insignificantly elevated level of *miR-9* in the blood of pT2 patients after operation; however, Yang et al. report insignificantly higher *miR-9* expression in T1 patients in comparison to a pooled T2-T4 group^47^. Our study population presented no significant dependencies between miRNA expression and tumour progression, probably because a large proportion of the cohort lacked local or adjacent metastasis, but previous studies suggest that *mir-9* plays a role in lung cancer invasiveness^46–48^.

As so far only a few papers have been published in relation to the topic of miRNA expression level changes depending on the histopathological type of NSCLC. Our study revealed higher *miR-9* expression level in AC in comparison to SCC.

### Role of miR-122 in NSCLC

In our study, we also analyzed expression level of *miR-122* in patients with diagnosed NSCLC. *MiR-122* is known as a cell proliferation inhibitor in multiple cancer cells, including NSCLC^19^. Interestingly Li et al. report upregulation of *miR-122* in exosomes of NSCLC^49^, suggesting that it may be used as a potential circulating biomarker for the diagnosis of NSCLC. Studies assessing the detailed roles or potential application of *miR-122* remain at an early stage.

In the present study, higher expression of *miR-122* was observed in NSCLC blood samples with elevated transcription level observed in SCC compared to AC. Although, as so far, has not been published data focus on evaluation in the blood expression level of *miR-122* in NSCLC patients. Others described downregulation of *miR-122* in AC cell lines^50^.

The *miR-122* expression level was insignificantly higher in pT3 samples than pT1 at both study time points. However, similar studies analysing samples before and after lung cancer resection has not been conducted, and there are no similar results comparing similar dependencies associated with *miR-122*.

Some studies have examined the relationship between *miR-122* expression, the *EGFR* pathway and cigarette addiction history in NSCLC patients^51,52^. It has been proposed that *miR-122* may play a role in the formation of AC^53^ and measuring plasma levels of *miR-122* may be useful in selection AC *EGFR* mutant patients^51^. Significantly downregulated expression of *miR-122* associated with EGFR gene mutation has been reported in a study assessing plasma and surgically-resected tumour tissues from male Japanese smokers with AC^52^. In our present work, *miR-122* expression was decreased in heavy smokers (PYs > 40). No significant correlations could be obtained with the *miR-122* analysis, perhaps because the majority of our population were those smoking < 40 PY and with SCC. Our findings did not identify the *EGFR* mutation status.

### Dependency between examined molecules

No significant correlations were found between the levels of selected miRNAs and interleukins. The data concerning the relationships between the analysed miRNAs and the chosen inflammatory interleukins used in the present study were obtained from bioinformatics analyses. It is possible that no correlations observed in our study may be related to the participation of other genes and target pathways for selected miRNAs. For example, *miR-9* has been found to target eukaryotic translation initiation factor 5A2 (eIF5A2)^46^, and to promote the proliferation, metastasis and invasion of NSCLC cells by down-regulating *TGFBR2* expression^48^.

Similarly, *miR-122* has been found to influence cyclin G1 or serum response factor, and insulin-like growth factor 1 receptor (IGF-1R)^54–56^. *MiR-122* also influence the survival of patients with AC by targeting these genes or by acting on the *GSK-3b–C/EBPa–miR-122–IGF-1R* regulatory pathway^57^. However, our findings do not contradict previous findings regarding the influence of *miR-9* and *miR-122* on inflammatory process in lung carcinogenesis. Further investigations, including both a larger group and a comparison of miRNA expression in tumour tissue and peripheral blood, including NSCLC lines, are required.

## Conclusions

- Increased expression of *IL-1β* and *IL-6* was found in both tumour and adjacent tissue, confirming the role of inflammatory processes in lung carcinogenesis.
- Significantly higher expression of the studied cytokines was observed in non-cancerous tissue in the surgical margin vs tumour, which may reflect the impact of other prevailing mechanisms (e.g. epigenetic ones) in primary lesions.
- Significantly variation in *IL-17* expression between AC and SCC may suggest it has a possible role in differentiation of NSCLC histopathological subtypes.
- Upregulated expression of *IL-6* in tumour samples among patients with ≤ 40 PYs vs. > 40 PYs demonstrate the impact of smoking on tumourigenesis
- No correlations were found between *miR-9* and *miR-122* and inflammatory processes—mediated via the studied cytokines—in lung carcinogenesis.
- The examined miRNAs do not appear to be potential molecular markers in lung carcinogenesis.

### Materials and methods

The study group comprised 19 women (mean age: 62.1, SD: 8.8 years) and 20 men (mean age: 64.8, SD: 8.3 years) of the Department of Thoracic Surgery, General and Oncologic Surgery, Medical University of Lodz, Poland, admitted in the period of July 2016–June 2017. Relevant clinical and pathological characteristics of all patients with NSCLC included in this study are summarized in Table 3.

**Table 3:** Histopathological summary of NSCLC samples. Legend as follows: *NSCLC non-small cell lung cancer, **AJCC American Joint Committee on Cancer Staging, ***pTNM post-operative Tumour Node Metastasis classification.

Analysed variables		Sample (n = 39)
Gender	Man	20 (51%)
	Woman	19 (49%)
Histopathologic type of NSCLC*	Squamous cell carcinoma (SCC)	25 (64%)
	Non-squamous cell carcinoma (NSCC)	14 (36%)
	*Adenocarcinoma (AC)*	*14 (36%)*
	*Large cell carcinoma (LCC)*	*0 (0%)*
AJCC**	AJCC IA/IB	18 (46%)
	AJCC IIA/IIB	17 (44%)
	AJCC III A/IIIB	4 (10%)
pTNM ***	T1	4 (10%)
	T2	32 (82%)
	T3-4	3 (8%)
Cigarette addiction	Active smokers	20 (51%)
	Former smokers	13 (33%)
	Non-smokers	1 (2%)
	Missing data	5(13%)
Pack years (PYs)	do 40 PYs	12 (31%)
	≥ 40 PYs	23 (59%)
	No data	4(10%)

All patients had histopathologically-confirmed primary NSCLCs. They were treated neither with chemo- nor radiotherapy. All patients underwent pulmonectomy or lobectomy, during which, tumour tissues (100–150 mg) and non-cancerous macroscopically unchanged tissue (100 mg) were taken from the surgical margin. These probes were immediately suspended in RNAlater buffer (Qiagen; Hilden, Germany) and frozen at − 80 °C. In addition, blood samples were collected from each patient on admission to hospital and 2 days after surgery to determine miRNA expression levels.

The relative expression of the studied samples was assessed using the comparative ΔΔCT method (TaqMan Relative Quantification Assay software; Applied Biosystems, Carlsbad, CA) and presented as relative expression (RQ) value, adjusted to ACTB (endogenous control) expression level. RNA isolated from normal lung tissue (Human Lung Total RNA, Ambion; Life Technologies, Carlsbad, CA) served as calibrator sample for which RQ = 1. RNAs obtained from macroscopically unchanged lung tissues served as a control group.

The study was funded by the scientific grant of Medical University in Lodz for Young Scientists, No.: 502–03/1–151-05/502-14-276. Its protocol was approved by the Ethical Committee of the Medical University of Lodz, Poland, agreement no. RNN/89/15/KE. All participating patients were fully informed. Written informed consent was obtained from each patient. All methods were carried out in accordance with relevant guidelines and regulations.

### MiRNA selection

After careful literature analysis we have selected *miR-9* (targeting *IL-6*^58,59^) and *miR-122* (targeting expression level of both *IL-1β*^17^ and *IL-6*^18^*)* for investigation in our research. The dependency for *miR-9* was confirmed using miRTarBase^12^.

### MiRNA analysis

**Figure 3:**
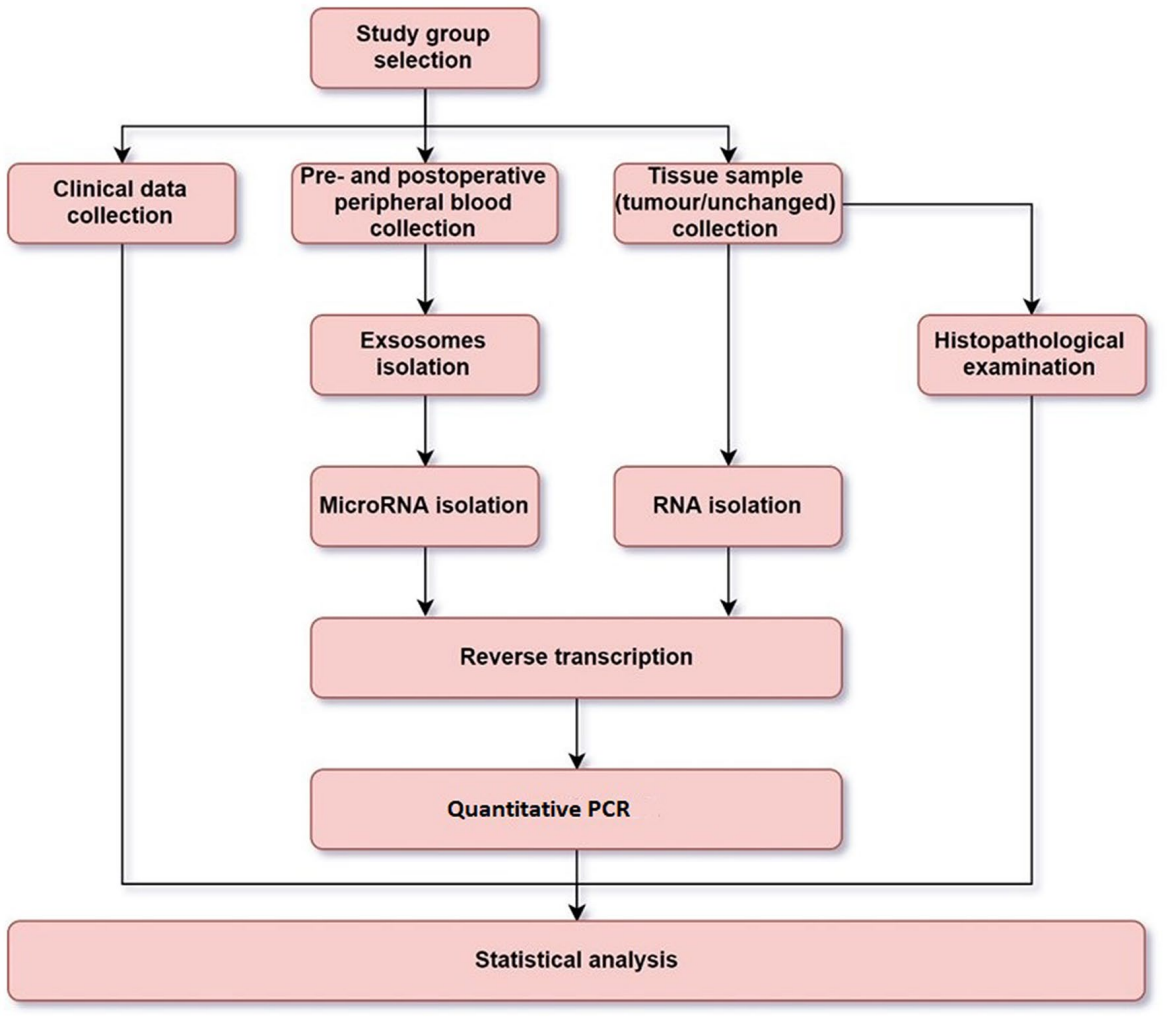
The study flowchart.

The first step in obtaining miRNA is isolation of exosomes from the serum stored at − 80 °C (Fig. 3). Isolation was performed using Total Exosome Isolation Reagent (from serum) (Cat. No. 4478360, Invitrogen). Following this, RNA (including miRNA) was isolated from exosomes by use of Total Exosome RNA and Protein Isolation Kit (Cat. No.: 4478545, Invitrogen). All procedures were followed according to the manufacturer’s protocol. Reverse transcription was then performed using a High-Capacity cDNA Reverse Transcription Kit with RNase Inhibitor (cat. No. 4374966, Applied Biosystem) and dedicated primers in a thermocycler (Eppendorf, Germany). Total reaction volume was equal to 15 μl: 10 μl of MasterMix and 5 μl of total RNA. The MasterMix comprised: dNTP Mix (0.15 μl), MultiScribe Reverse Transcriptase (1 μl), 10 × RT Buffer (1.5 μl), RNase Inhibitor (0.19 μl), Nuclease-free water (4.16 μl) and dedicated primers (3 μl). After the reaction, the product was diluted with 43.5 μl of Nuclease-free water. qPCR was performed in the 7900HT Fast Real-Time PCR System. The 10 µl of PCR mixture contained: 4.5 μl of diluted RT product, 5 μl of KAPA PROBE FAST qPCR Kit, ROX (ABI Prism) (cat. No. KK4706, Kapa Biosystem) and 0.5 μl of miRNA probes. The calibrator was healthy volunteer peripheral blood sample. The analysis, performed in DataAssist v3.01, was based on the global normalization strategy.

### Gene analysis

Firstly, the total RNA was isolated from the homogenized tissue using a Qiagen RNA mini Kit (Cat. No. 74104, QIAGEN) according to the manufacturer’s protocol. RT-PCR was then performed using a High-Capacity cDNA Reverse Transcription Kit (cat. No. 4368814, Applied Biosystem) in a thermocycler (Eppendorf, Germany). Total reaction volume was equal to 20 μl: 10 μl of MasterMix and 10 μl of total RNA. The MasterMix comprised: dNTP Mix (0.8 μl), MultiScribe Reverse Transcriptase (1 μl), 10 × RT Buffer (2 μl), RNase Inhibitor (1 μl), 10XRT Random Primers (2 μl), and Nuclease-free water (4.16 μl). Q-PCR was performed in 7900HT Fast Real-Time PCR System. The 10 µl of PCR mixture contained: 1.0 μl of cDNA, 5 μl of KAPA PROBE FAST qPCR Kit, ROX (ABI Prism) (cat. No. KK4706, Kapa Biosystem), 0.5 μl of dedicated probes and 3.5 μl of RNAse-free water. RNA isolated from normal lung tissue (Human Lung Total RNA, Ambion, USA) was used as the calibrator. The analysis was performed in DataAssist v3.01 according to ΔΔCt.

### Statistical analysis

Statistical analysis was performed using Statistica 13.1 PL (StatSoft). When comparing groups without a normal distribution, the Kruskal–Wallis, Mann–Whitney U-test, Wilcoxon tests and the Spearman’s rank correlation were performed. For data with a normal distribution, parametric equivalents were chosen. A p-value less than 0.05 was considered as statistically significant unless otherwise stated. Results are presented as mean ± SD for normally distributed data, and median with IQR in other cases.

## Strengths and weaknesses of the study

The strengths of this study are:

- the prospective design;
- inclusion of the NSCLC patients that were not treated with potentially mutagenic chemotherapy or radiotherapy prior to the surgery;
- analysis in the most common NSCLC subtypes: AC and SCC;
- analysis of microRNAs expression before and after surgical treatment;

The weaknesses of the study are:

- The mRNA expression of the tested interleukins was not assessed at protein level;
- Small study group size;
- High dropout rate.
